# Salivary Biomarker Profile in Periodontal Diseases: A Cross-Sectional Study on Leptin, Adiponectin, and Calprotectin

**DOI:** 10.3390/diagnostics15222822

**Published:** 2025-11-07

**Authors:** Ali Batuhan Bayırlı, Mehmetcan Uytun, Fulden Cantaş Türkiş, Ercan Saruhan, Hüseyin Gencay Keceli

**Affiliations:** 1 Department of Periodontology, School of Dentistry, Mugla Sıtkı Kocman University, 48000 Mugla, Türkiye; mehmetcanuytun@mu.edu.tr; 2 Department of Biostatistics, School of Medicine, Mugla Sıtkı Kocman University, 48000 Mugla, Türkiye; fuldencantas@mu.edu.tr; 3 Department of Biochemistry, School of Medicine, Mugla Sıtkı Kocman University, 48000 Mugla, Türkiye; ercansaruhan@mu.edu.tr; 4 Department of Periodontology, School of Dentistry, Hacettepe University, 06800 Ankara, Türkiye; monsieur_gencay@yahoo.com

**Keywords:** adiponectin, calprotectin, gingivitis, leptin, periodontitis, saliva

## Abstract

**Background/Objectives**: This study aimed to evaluate salivary leptin, adiponectin, and calprotectin levels and to investigate the associations among these biomarkers in periodontally healthy individuals, as well as in patients with gingivitis and periodontitis. **Methods**: A total of 165 participants were included: 55 periodontally healthy individuals, 55 with gingivitis, and 55 with periodontitis. Unstimulated saliva was collected via passive drool, and salivary leptin, adiponectin, and calprotectin levels were biochemically quantified using enzyme-linked immunosorbent assay. **Results**: Salivary leptin levels were significantly lower in the periodontally healthy group than those in the gingivitis and periodontitis groups, whereas adiponectin levels were reduced in the periodontitis group than in the periodontally healthy and gingivitis groups (*p* < 0.05). Salivary calprotectin levels differed significantly among groups, highest in the periodontitis group, followed by the gingivitis and periodontally healthy groups (*p* < 0.05). Salivary leptin and calprotectin levels demonstrated significant positive correlations with all clinical periodontal parameters, while adiponectin levels were negatively correlated (*p* < 0.05). Receiver operating characteristic and logistic regression analyses identified salivary leptin, calprotectin, and adiponectin levels as significant biomarkers for distinguishing periodontal health, gingivitis, and periodontitis (*p* < 0.05). **Conclusions**: These findings suggest salivary leptin, calprotectin, and adiponectin may serve as biomarkers and potential risk predictors of periodontal disease.

## 1. Introduction

Oral health is closely linked to systemic diseases. Therefore, regular dental examinations and early diagnosis of oral diseases are vital for maintaining oral and overall systemic health [1]. Systemic conditions that promote inflammation, such as obesity, are significant risk factors for the development of periodontal diseases [2]. The World Health Organization defines obesity as the abnormal or excessive accumulation of adipose tissue that may adversely impact health [3]. An imbalance between energy intake and expenditure leads to obesity, linked to chronic diseases including diabetes, cardiovascular diseases, and hypertension, and periodontal diseases [4,5,6].

Adipokines are biochemical mediators that contribute to the development of obesity and exhibit altered expression throughout this process [7]. Moreover, these adipokines have been implicated in the pathogenesis of several inflammatory diseases, including type 2 diabetes, cardiovascular diseases, and rheumatoid arthritis [8,9]. Recent studies have suggested that adipokines are associated with periodontal diseases [10,11,12]. Leptin is an adipokine hormone secreted by adipose tissue that regulates energy metabolism while modulating inflammatory responses. Elevated leptin levels are commonly observed in individuals with obesity and type 2 diabetes. However, over time, leptin resistance impairs appetite regulation and exacerbates systemic inflammation [13,14]. A bidirectional relationship between periodontal disease pathogenesis and serum leptin levels has been demonstrated [10]. Moreover, adiponectin is an adipokine known for its anti-inflammatory and insulin-sensitizing properties [15]. Additionally, adiponectin is primarily synthesized by the white adipose tissue [16]. Reduced adiponectin levels have been reported to increase the risk of systemic inflammation and periodontal disease [11,17]. Calprotectin is a heterodimeric complex of calcium-binding proteins secreted into circulation by monocytes and neutrophils, playing a key role in inhibiting microbial growth during inflammatory processes and serving as a well-established biomarker of inflammation [18]. Furthermore, calprotectin exerts its antimicrobial activity via zinc sequestration [19]. Elevated serum calprotectin levels have been implicated in systemic inflammatory diseases, including inflammatory bowel disease and rheumatoid arthritis [19,20]. Furthermore, increased calprotectin levels in gingival crevicular fluid (GCF) and saliva have been observed in periodontal disease [12,21]. Assessing the levels of these biomarkers in periodontal tissues may provide valuable insights into the systemic implications of periodontal diseases.

In this context, saliva-based diagnostic approaches offer several clinical advantages, including non-invasiveness, ease of collection, and the potential for real-time chairside monitoring. These characteristics make saliva an attractive medium for detecting inflammatory and metabolic biomarkers associated with periodontal disease, thereby facilitating early diagnosis and personalized treatment planning [22,23,24].

Leptin, adiponectin, and calprotectin levels have been hypothesized to vary across different stages of periodontal disease. Consistent with the increased inflammatory response observed in periodontal conditions, such as gingivitis and periodontitis, elevated levels of leptin and calprotectin, along with decreased levels of adiponectin, an anti-inflammatory adipokine, are anticipated. Although previous studies have evaluated salivary levels of leptin, calprotectin, and adiponectin in systemic and periodontal diseases, those comparing the levels of these biomarkers across periodontal health, gingivitis, and periodontitis within the same population remain limited [25,26,27]. The lack of a comprehensive study evaluating all three biomarkers in relation to periodontal status within the same sample group highlights a significant gap in the existing literature. A deeper understanding of how these adipokines and inflammatory mediators interact across the spectrum of periodontal inflammation could clarify the molecular crosstalk between metabolic and immune pathways. Establishing such biomarker-based profiles would enhance the pathophysiological understanding of periodontal disease and support the development of non-invasive diagnostic strategies. To the best of our knowledge, this is the first study to simultaneously assess salivary leptin, adiponectin, and calprotectin levels in non-obese, systemically healthy adults across different periodontal conditions. Furthermore, the inclusion of advanced statistical approaches enabled the assessment of diagnostic performance and predictive value of these biomarkers. This study aimed to evaluate the effects of salivary leptin, adiponectin, and calprotectin levels on periodontal tissues and to assess the relationships among these biomarkers. The null hypothesis (H_0_) stated that salivary leptin, adiponectin, and calprotectin levels do not differ among individuals with periodontal health, gingivitis, and periodontitis.

## 2. Materials and Methods

### 2.1. Study Design and Participants

This study was approved by the Non-Interventional Clinical Research Ethics Committee of İzmir Bakırçay University on 12 February 2025 (Approval No: 2032) and performed in accordance with the principles of the Declaration of Helsinki. All individuals were fully informed about the study’s aim and procedures, and written informed consent was obtained. The study was performed following the Strengthening the Reporting of Observational Studies in Epidemiology guidelines. Participants were consecutively recruited between March 2025 and May 2025 from individuals presenting to the Department of Periodontology, Faculty of Dentistry, Muğla Sıtkı Koçman University. Clinical and radiographic examinations were performed at the Department of Periodontology, Faculty of Dentistry, Muğla Sıtkı Koçman University. A total of 165 participants who met the following criteria were included in the study: (i) age between 25 and 40 years, (ii) absence of any systemic disease, (iii) no regular use of systemic or topical medications, (iv) no periodontal treatment within the past 6 months; and (v) non-smokers. Participants aged 25–40 years were included, as this range minimizes age-related variability in periodontal destruction, hormonal fluctuations, and systemic comorbidities. This selection ensured a metabolically stable and homogeneous adult population, thereby improving the accuracy and reliability of salivary biomarker comparisons across periodontal health, gingivitis, and periodontitis groups [28,29]. Individuals meeting any of the following criteria were excluded from the study: (i) presence of systemic inflammatory diseases including diabetes, cardiovascular diseases, rheumatoid arthritis, or inflammatory bowel disease; (ii) diagnosis of obesity; (iii) pregnancy or lactation; (iv) history of immunosuppressive therapy or steroid use; (v) clinical signs of active infection, such as periodontal abscess or other acute periodontal conditions; and (vi) use of antibiotics or anti-inflammatory medications within the past 3 months. This three-month antibiotic-free interval was selected to avoid residual alterations in the oral microbiome and inflammatory status that could influence salivary biomarker levels, particularly leptin, adiponectin, and calprotectin. Similar exclusion criteria have been applied in recent high-impact periodontal studies to ensure a stable microbial and host-response baseline [30,31]. Participants meeting the inclusion criteria were included as periodontally healthy, gingivitis, or Stage III periodontitis. Only patients diagnosed with Stage III periodontitis were included in the periodontitis category, while patients with other stages of periodontitis were excluded.

### 2.2. Study Groups

Eligible participants were consecutively recruited from individuals presenting to the Faculty of Dentistry, Muğla Sıtkı Koçman University. Participants were categorized into three groups based on their periodontal status: periodontally healthy, gingivitis, and periodontitis. Periodontal examination included assessment of the plaque index (PI), gingival index (GI), bleeding on probing (BOP), probing pocket depth (PPD), and clinical attachment loss (CAL). The periodontal status of participants in each study group was classified according to the criteria established by the European Federation of Periodontology and American Academy of Periodontology in 2017 [32,33]. The inclusion criteria for each group are as follows:

Periodontally healthy group (*n* = 55): Included individuals with CAL ≤ 1 mm and PPD ≤ 3 mm on all teeth, no radiographic bone loss, and BOP < 10%.

Gingivitis group (*n* = 55): Included individuals with CAL ≤ 1 mm and PPD ≤ 3 mm on all teeth, no radiographic bone loss, and BOP ≥ 10%.

Periodontitis group (*n* = 55): Included patients with stage III periodontitis, no more than four teeth lost due to periodontal reasons, CAL ≥ 5 mm and PPD ≥ 6 mm on at least one tooth, and radiographic bone loss extending to the middle or apical third of the root.

### 2.3. Periodontal Examination

Comprehensive clinical and radiographic assessments were performed to evaluate the periodontal status of all participants. Clinical measurements were obtained at six sites per tooth using a Williams periodontal probe (Hu-Friedy, Chicago, IL, USA). Third molars were excluded from the periodontal examination to maintain standardization across participants. For each participant, the mean values of PI, GI, BOP, PPD, and CAL were calculated based on all measured sites. All the measurements were conducted by a single periodontist (A.B.B.). Before data collection, intra-examiner calibration was performed for 10 patients with stage III periodontitis. Intra-examiner reliability was assessed using the intraclass correlation coefficient, yielding values of 0.94 for PPD and 0.93 for CAL, indicating excellent measurement reliability.

### 2.4. Saliva Sample Acquisition

For biochemical analyses, unstimulated saliva samples were noninvasively collected using the passive drool technique [27], with one sample obtained per participant across all groups. To ensure standardization, all unstimulated saliva samples were collected in the morning into Eppendorf tubes. Before providing 3–5 mL of saliva, the participants rinsed their mouths with water for 2 min to remove food debris. The collected samples were centrifuged at 1000× *g* for 10 min. The supernatants were transferred to Eppendorf tubes and stored at −80 °C until biochemical analysis.

For biochemical analyses, unstimulated saliva samples were noninvasively collected using the passive drool technique [34], with one sample obtained per participant across all groups. To ensure standardization, all unstimulated saliva samples were collected in the morning into Eppendorf tubes. Participants were instructed to refrain from eating, drinking (except water), chewing gum, and toothbrushing for at least one hour before sampling to minimize variability in salivary biomarker concentrations [35]. Before providing 3–5 mL of saliva, the participants rinsed their mouths with water for 2 min to remove food debris. The collected samples were centrifuged at 1000× *g* for 10 min using a Hettich Universal 320R centrifuge (Hettich Zentrifugen, Tuttlingen, Germany). The supernatants were transferred to Eppendorf tubes and stored at −80 °C until biochemical analysis.

### 2.5. Leptin Measurements

Salivary leptin concentrations were assessed using a human leptin enzyme-linked immunosorbent assay (ELISA) kit (Cat# E1559Hu, BT-laboratory, Shanghai, China) according to the manufacturer’s instructions. Measurements were carried out using an ELISA plate reader (Multiskan GO microplate reader, Thermo Fisher Scientific, Waltham, MA, USA). The leptin assay had a sensitivity of 0.021 ng/mL, with inter- and intra-assay coefficients of variation below 10%.

### 2.6. Adiponectin Measurements

Salivary adiponectin concentrations were examined using a human adiponectin ELISA kit (Cat# E4685Hu, BT-laboratory, Shanghai, China) according to the manufacturer’s instructions. Measurements were carried out using an ELISA plate reader (Multiskan GO microplate reader, Thermo Fisher Scientific, Waltham, MA, USA). The adiponectin assay sensitivity was 0.11 ng/mL with inter- and intra-assay coefficients of variation of below 10%.

### 2.7. Calprotectin Measurements

Salivary calprotectin concentrations were evaluated using a human calprotectin ELISA kit (Cat# E4010Hu, BT-laboratory, Shanghai, China) based on the manufacturer’s instructions. Measurements were performed using an ELISA plate reader (Multiskan GO microplate reader, Thermo Fisher Scientific, Waltham, MA, USA). The sensitivity of the calprotectin assay was 1.67 ng/mL with inter- and intra-assay coefficients of variation below 10%.

### 2.8. Statistical Analysis

All statistical analyses were performed using IBM SPSS Statistics for Windows, Version 27.0 (IBM Corp., Armonk, NY, USA) and MedCalc Statistical Software version 20.218 (MedCalc Software Ltd., Ostend, Belgium). The normality of continuous variables was assessed using the Kolmogorov–Smirnov test. Descriptive statistics were presented as median and interquartile range for non-normally distributed variables and as counts with percentages (*n*,%) for categorical variables. Group comparisons for continuous variables were performed using the Kruskal–Wallis test, followed by post hoc pairwise comparisons with Dunn–Bonferroni correction. The chi-square test was employed to compare categorical variables. Receiver operating characteristic (ROC) curve analysis evaluated the diagnostic performance of leptin, calprotectin, and adiponectin in distinguishing between periodontal states (healthy, gingivitis, and periodontitis). Comparisons of area under the curve (AUC) values between biomarkers were performed using DeLong’s test. The optimal cutoff values for each biomarker were determined based on the Youden index, which maximized the sum of sensitivity and specificity. Biomarkers were dichotomized according to the optimal thresholds obtained from the ROC analysis. Univariate and multiple binary logistic regression (LR) analyses assessed the associations between biomarker levels and periodontal disease status. Odds ratios (ORs) with 95% confidence intervals (CIs) and *p*-values were also calculated. Variables with *p* < 0.05 in univariate analysis were included in the multiple models using the enter method. To evaluate potential multicollinearity among the independent variables, variance inflation factor (VIF) values were examined, and all values were found to be <2, indicating an acceptable level of collinearity. Spearman’s correlation coefficients were calculated to explore the relationships between the clinical periodontal parameters and biomarker levels. The correlation matrix was visualized as a heat map to illustrate the strengths and direction of association. A *p*-value < 0.05 was considered statistically significant throughout all analyses.

Power analysis: A literature review revealed no studies simultaneously evaluating leptin, adiponectin, and calprotectin in relation to periodontal status. Therefore, no previous studies could be directly referenced in planning the present research. Accordingly, an a priori power analysis was performed. Power analysis was performed using one-way analysis of variance, with an effect size of 0.25, classified as medium according to Cohen’s classification [36]. The type I error probability (α) was set at 0.05, and statistical power (1 − β) at 0.80. Based on these parameters, a minimum of 53 participants per group (total *n* = 159) was required. Power analysis was performed using G*Power software (version 3.1.2).

## 3. Results

A total of 165 individuals were included: 55 periodontally healthy, 55 with gingivitis, and 55 with periodontitis. In the periodontally healthy group, 27 (49.1%) were female and 28 (50.9%) male; in the gingivitis group, 27 (49.1%) female and 28 (50.9%) male; and in the periodontitis group, 28 (50.9%) female and 27 (49.1%) male (*p* = 0.999, Cramer’s V = 0.017). The median ages were 33 years (range 24–38) for the healthy and gingivitis groups, and 33 years (range 30–38) for the periodontitis group. No statistically significant differences in age were observed between the groups (*p* = 0.466, η^2^ = 0.010) (Table 1).

Statistically significant differences were noted between the groups for all clinical periodontal parameters (*p* < 0.001). As expected, the lowest values for all clinical parameters were observed in the healthy group and the highest in the periodontitis group. According to the post hoc test results, PI, GI, and BOP values differed significantly across all groups (*p* < 0.001 for all pairwise comparisons) with large effect sizes (η^2^ = 0.575, 0.603, and 0.790, respectively) (Table 1). Regarding PPD and CAL, the periodontitis group demonstrated significantly higher values compared to those in the other groups (*p* < 0.001 for all pairwise comparisons), also showing large effect sizes (η^2^ = 0.691 and 0.664, respectively) (Table 1).

Significant differences were also observed among the groups in terms of salivary biochemical marker levels (*p* < 0.001). Salivary leptin levels were significantly decreased in the periodontally healthy group compared to the levels in both the gingivitis and periodontitis groups (*p* = 0.037 and *p* < 0.001, respectively; η^2^ = 0.110). The periodontitis group demonstrated significantly lower salivary adiponectin levels than those observed in the periodontally healthy and gingivitis groups (*p* < 0.001 for all pairwise comparisons; η^2^ = 0.225). Salivary calprotectin levels also differed significantly among all groups, showing a progressive increase from the healthy to gingivitis and periodontitis groups, with pairwise differences between all groups (*p* = 0.004, *p* < 0.001, and *p* = 0.012, respectively; η^2^ = 0.215). In addition, no statistically significant differences were observed between the gingivitis and periodontitis groups regarding salivary leptin levels or between the healthy and gingivitis groups for adiponectin levels (*p* > 0.05 for all pairwise comparisons) (Table 1).

**Figure 1:**
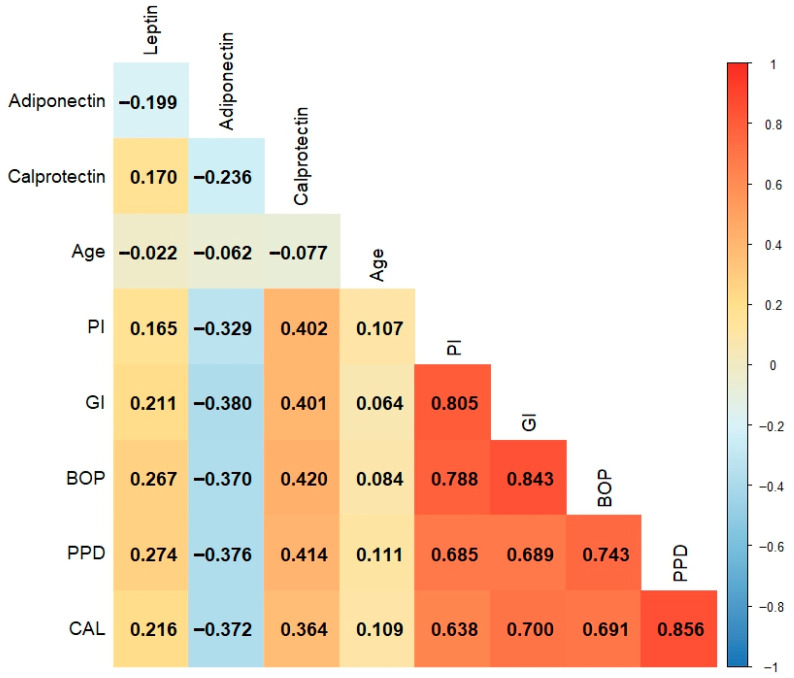
Correlation analysis among clinical, biochemical, and anthropometric variables. Spearman’s correlation coefficients are visualized using a color gradient, with red indicating strong positive correlations, while blue suggests strong negative correlations. Only statistically significant correlations (*p* < 0.05) are considered for the interpretation. For clarity, only the lower triangle of the symmetrical matrix is presented. CAL, clinical attachment loss; BOP, bleeding on probing; GI, gingival index; PPD, probing pocket depth; PI, plaque index.

Associations between salivary leptin, adiponectin, and calprotectin levels and clinical periodontal parameters were evaluated individually. Spearman correlation analyses revealed that salivary leptin levels were strongly positively correlated with the PI, GI, BOP, PPD, and CAL scores (*p* < 0.05). Similarly, salivary calprotectin levels were significantly and positively correlated with all clinical periodontal parameters (*p* < 0.001). Conversely, salivary adiponectin levels were significantly negatively correlated with all clinical periodontal parameters (*p* < 0.001). Moreover, salivary leptin levels demonstrated a significant positive correlation with calprotectin levels (*p* = 0.029) and a negative correlation with adiponectin levels; both correlations were statistically significant (*p* = 0.010) (Figure 1).

### 3.1. Healthy Group Versus Gingivitis Group

**Figure 2:**
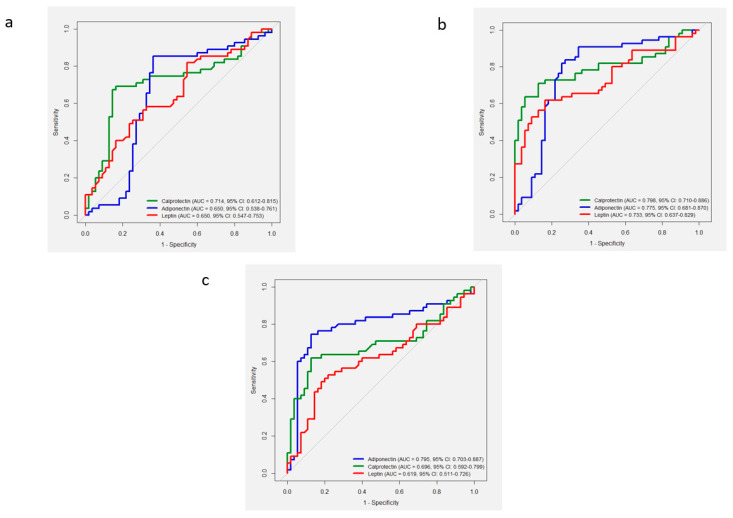
Comparison of receiver operating characteristic (ROC) curves of salivary leptin, adiponectin, and calprotectin in distinguishing different periodontal conditions. (**a**) Comparison of ROC curves of salivary biomarkers for differentiating healthy and gingivitis groups. (**b**) Comparison of ROC curves of salivary biomarkers for differentiating healthy and periodontitis groups. (**c**) Comparison of ROC curves of salivary biomarkers for distinguishing the gingivitis and periodontitis groups.

According to the results of the ROC analysis, leptin, calprotectin, and adiponectin were identified as significant biomarkers for the diagnosis of gingivitis (*p* < 0.01). However, multiple comparisons revealed no significant differences in AUC among the three biomarkers (*p* > 0.05) (Table 2) (Figure 2a).

In the multiple logistic regression models, leptin, adiponectin, and calprotectin levels were included as independent variables, while age and sex were not entered due to their homogeneity across groups. According to the results of the univariate LR analysis, elevated leptin and calprotectin levels and reduced adiponectin levels were significantly associated with the presence of gingivitis (*p* < 0.01). In the multiple LR model, calprotectin and adiponectin levels were significantly associated with gingivitis (*p* < 0.001). In contrast, the effect of leptin levels on gingivitis was not statistically significant in the multiple LR analysis (*p* > 0.05) (Table 3).

### 3.2. Healthy Group Versus Periodontitis Group

According to the results of the ROC analysis, leptin, calprotectin, and adiponectin were identified as significant biomarkers for the diagnosis of periodontitis (*p* < 0.001). However, multiple comparisons revealed no statistically significant differences in the AUC of the three biomarkers (*p* > 0.05) (Table 2) (Figure 2b).

Univariate LR analysis demonstrated that higher leptin and calprotectin levels were significantly associated with periodontitis. Similarly, decreased adiponectin levels were significantly associated with periodontitis (*p* < 0.001). In the multiple LR model, leptin, adiponectin, and calprotectin levels were significantly associated with periodontitis (*p* < 0.001) (Table 3).

### 3.3. Gingivitis Group Versus Periodontitis Group

According to the results of the ROC analysis, leptin, calprotectin, and adiponectin served as significant biomarkers for the diagnosis of periodontitis (*p* < 0.05) (Table 2) (Figure 2c). Comparison of AUC values revealed that adiponectin had a significantly higher AUC than that noted with leptin (*p* = 0.006).

According to the results of the univariate LR analysis, high leptin and calprotectin levels and low adiponectin levels were significantly associated with periodontitis (*p* < 0.01). In the multiple LR model, the effects of calprotectin and adiponectin levels on periodontitis remained significant (*p* < 0.001). Conversely, leptin levels were not significantly associated with periodontitis in multiple analysis (*p* > 0.05) (Table 3).

## 4. Discussion

In this study, salivary leptin, adiponectin, and calprotectin levels were compared between periodontally healthy, gingivitis, and periodontitis groups. Moreover, the findings were evaluated in relation to clinical periodontal parameters. The null hypothesis stating that salivary leptin, adiponectin, and calprotectin levels do not differ among individuals with periodontal health, gingivitis, and periodontitis was rejected, as significant intergroup differences were observed for all biomarkers. Our main findings indicate that salivary leptin levels increase with disease severity, supporting the role of this adipokine in the inflammatory cascade of periodontal disease. This increase is consistent with the findings of the existing literature, indicating that leptin is involved not only in energy metabolism but also actively participates in the inflammatory process [10,11,12,13,14]. Leptin potentially contributes to connective tissue breakdown by enhancing the release of proinflammatory cytokines. In particular, leptin stimulates the production of interleukin (IL)-6 and tumor necrosis factor (TNF)-α through its effects on monocytes and macrophages, thereby promoting inflammation. Indeed, previous studies have demonstrated that leptin can induce the expression of IL-6, TNF-α, and matrix metalloproteinases (MMPs) in periodontal tissues [37,38,39]. Consistent with these outcomes, our results suggest that increased salivary leptin levels are associated with periodontal inflammation and tissue destruction. Similarly, to the distribution pattern of leptin, salivary calprotectin levels significantly increased with disease severity. Calprotectin is primarily secreted by neutrophils, but may also be produced by monocytes. Although calprotectin plays a crucial role in antimicrobial defense, excessive accumulation contributes to local inflammation, indirectly promoting tissue destruction [40,41]. The notably elevated calprotectin levels in the periodontitis group reflect its strong association with neutrophil activity and tissue breakdown [42]. Previous studies have demonstrated that calprotectin levels are markedly elevated in both the saliva and GCF of individuals with gingivitis and periodontitis [12,42]. Unlike leptin and calprotectin, the anti-inflammatory adipokine adiponectin was highest in the healthy group and significantly lower in the periodontitis group. This finding supports the protective role of adiponectin in periodontal tissues and suggests that anti-inflammatory defense mechanisms may diminish with the progression of the disease. Similarly, previous studies have reported high adiponectin levels in the saliva and gingival tissues of periodontally healthy individuals [43,44]. Adiponectin plays a regulatory role in bone metabolism, pro-inflammatory cytokine production, and inflammatory responses in periodontal tissues, contributing to the pathogenesis of periodontitis. Adipokine effects on periodontal tissues may vary between both protective and pro-inflammatory, depending on factors such as the local tissue microenvironment, concentration, and interactions with other immune mediators [11,45]. Moreover, another adipokine, resistin, has been implicated in the pathogenesis of periodontal disease. Elevated resistin levels have been reported in individuals with periodontitis, suggesting its potential role as a pro-inflammatory adipokine contributing to periodontal tissue inflammation and disease progression [46]. Beyond soft-tissue inflammation, recent reports describe fatty degenerative changes in jawbone marrow that may impair local microcirculation and limit regenerative capacity. Although not directly assessed in the present study, such marrow alterations could mechanistically link metabolic dysregulation to alveolar bone remodeling, potentially accelerating periodontal breakdown [47]. Mechanistically, dysregulation of adipokines may contribute to the chronic inflammatory milieu in periodontal tissues. Persistent elevation of pro-inflammatory adipokines can lead to receptor desensitization, as observed with leptin resistance, and amplify cytokine-driven neutrophil activation and oxidative stress, whereas anti-inflammatory adipokines such as adiponectin counteract these effects through NF-κB inhibition and redox modulation. Moreover, factors such as diet, hormonal fluctuations, and circadian rhythm may further influence salivary biomarker levels and should be considered in future research [48].

In our study, not only the group-level distributions of salivary leptin, adiponectin, and calprotectin levels, but also their relationships with clinical periodontal parameters were of particular importance in reflecting disease severity. Previous studies demonstrated that leptin, calprotectin, and adiponectin levels are associated with periodontal status and disease severity [12,43,49,50]. The correlations between these biomarkers and clinical parameters were also examined to provide a detailed evaluation of their association with periodontal disease severity. The correlations observed between salivary biomarker levels and clinical parameters reinforce their pathophysiological relevance, linking biochemical changes with tissue destruction and inflammation. These findings reflect associations observed within a cross-sectional framework and do not imply causal or temporal relationships. Furthermore, these levels exhibited significant diagnostic potential in distinguishing periodontal health from disease. Adiponectin exhibited the greatest discriminative capacity between gingivitis and periodontitis, underscoring its potential diagnostic relevance. Although all three biomarkers demonstrated significant diagnostic performance, the absence of substantial differences in AUC among leptin, adiponectin, and calprotectin in most comparisons suggests comparable diagnostic potential rather than distinct biomarker specificity. However, the relatively higher AUC of adiponectin in differentiating gingivitis from periodontitis may indicate greater sensitivity to disease progression. Collectively, these findings indicate that calprotectin and adiponectin exert independent influences on periodontal disease progression, whereas leptin appears to have a more limited role in multiple contexts. These findings support the notion that salivary leptin, calprotectin, and adiponectin levels have potential diagnostic and risk-predictive value for periodontal diseases, as previously reported in the literature [37,41,43]. However, it should be noted that these associations were identified within a cross-sectional framework and therefore reflect correlations observed at a single time point. Future longitudinal studies with repeated biomarker measurements are required to confirm their prognostic validity.

The positive correlation between salivary calprotectin and the clinical periodontal parameters supports its role as a marker of neutrophil-driven inflammation [42]. Although several studies have investigated the association between GCF calprotectin levels and periodontal status, those focusing on salivary calprotectin levels remain limited. A recent scoping review reported elevated calprotectin levels in GCF, saliva, and serum of individuals with periodontal disease compared to healthy controls [21]. These findings also suggest that both saliva and GCF may provide complementary biochemical insights into periodontal status. Gao et al. [12] reported that GCF and serum calprotectin levels were higher in the periodontitis group than in the healthy group. A statistically significant positive correlation was noted between GCF calprotectin levels and PPD. Similarly, another study with a comparable design reported higher GCF calprotectin levels in patients with periodontitis than in healthy individuals. Moreover, researchers have reported a significant reduction in GCF calprotectin levels following non-surgical periodontal treatment in a periodontitis group compared to baseline. Although salivary calprotectin levels also decreased following treatment, from 0.17 ± 0.12 μg/μL to 0.10 ± 0.09 μg/μL, this change was not statistically significant [51]. In a study by Holmström et al. [40], calprotectin levels were reported to be associated with periodontal parameters, with salivary calprotectin levels significantly higher in individuals with BOP > 20% and PPD ≥ 4 mm. Similarly, Haririan et al. [52] reported that salivary calprotectin levels were approximately 2.8 times higher (quotient of mean CI = 2.83) in individuals with periodontitis than in the levels in periodontally healthy individuals. Additionally, this biomarker exhibited a positive correlation with clinical periodontal parameters. Furthermore, experimental gingivitis models induced by plaque accumulation demonstrated significant increases in salivary calprotectin levels corresponding to plaque buildup [53].

Two studies that investigated the relationship between salivary leptin levels and periodontal status reported that the levels were higher in patients with periodontitis than in healthy individuals [49,50]. In another study, Jia et al. [54] investigated the biomarkers involved in lipid metabolism in obese patients with periodontitis and reported a positive association between serum leptin levels and periodontitis. Biomarker levels were also assessed in saliva. Saliva is the preferred sampling method due to its significant correlation with serum and plasma levels of leptin, adiponectin, and calprotectin, as well as its noninvasive nature and improved patient compliance [55,56,57]. Silva et al. [58] reported no correlation between salivary leptin levels and periodontal status in their study. Researchers have suggested that salivary leptin levels are primarily influenced by systemic rather than by periodontal inflammation. Additionally, leptin is secreted in oligomeric form by the salivary glands, which may have reduced solubility in saliva, potentially leading to lower measurable leptin concentrations [59,60]. Sales-Peres et al. [61] studied morbidly obese premenopausal females under the age of 35 years and identified no correlation between salivary leptin levels and periodontal parameters. This may be attributed to the small sample size and narrow, homogeneous characteristics of the study population. In contrast, our study included a large and relatively heterogeneous sample.

In our study, the significant negative correlation between salivary adiponectin levels and clinical periodontal parameters supports its role in suppressing periodontal inflammation. However, the findings regarding adiponectin levels in the literature remain inconsistent. Two studies on obese individuals with periodontitis reported conflicting results, one identified significantly lower GCF adiponectin levels in individuals with periodontitis compared to the levels in the healthy controls [62], while the other reported higher GCF adiponectin levels in females with periodontitis than in healthy females [63]. This discrepancy may be attributed to differences in sex distribution across studies, as adiponectin levels are known to vary according to sex [64]. The coexistence of obesity and periodontitis has been reported to increase salivary leptin levels, but it does not significantly affect adiponectin levels [65]. Furthermore, studies investigating the coexistence of diabetes mellitus and periodontitis discovered no significant association between salivary adiponectin levels and the presence or severity of periodontal disease [66,67]. However, other studies have reported that adiponectin levels decrease in the presence of periodontitis and are positively associated with periodontal health [43]. The rise in salivary adiponectin levels following periodontal treatment suggests that this biomarker has the potential for monitoring periodontal diseases [68,69]. Leptin, calprotectin, and adiponectin levels are influenced by various systemic factors, including obesity, diabetes mellitus, metabolic syndrome, and hormonal changes [10,42,64,70,71]. Therefore, only systemically healthy individuals were included in our study to accurately assess the independent effects of these biomarkers on periodontal disease. To the best of our knowledge, this is the first study to concurrently evaluate salivary leptin, adiponectin, and calprotectin levels in relation to periodontal health, gingivitis, and periodontitis, and to assess their diagnostic and predictive potential through combined correlation, logistic regression, and ROC analyses. This comprehensive approach provides novel insight into the collective biochemical mechanisms underlying periodontal inflammation and tissue destruction, thereby enhancing our understanding of saliva-based diagnostics in periodontal disease.

This study had several limitations. First, the cross-sectional design did not allow the establishment of causal relationships between biomarker levels and periodontal disease. In addition, the relatively limited sample size and single-center design restrict the generalizability of the findings to broad populations. Although the exclusion of obese individuals strengthened the internal validity of the present study by minimizing systemic metabolic confounders, this methodological choice may restrict the external generalizability of the findings to broader populations, particularly those with obesity-related inflammatory alterations. Additionally, the lack of standardized reference ranges for leptin, adiponectin, and calprotectin remains a major limitation in the clinical interpretation of the results. Moreover, although systemic and behavioral factors, such as diet and stress, may influence biomarker levels, they were not controlled in the present study. This study evaluated biomarker levels based solely on the current periodontal status, without assessing the effects of periodontal treatment. Another limitation was the lack of normalization of salivary biomarker concentrations by flow rate or total protein content, which could improve comparability across individuals. In addition to the biomarkers assessed, the inclusion of other parameters such as MMP-8, IL-1β, and TNF-α could provide comprehensive insights into the pathogenesis of periodontal disease. Future research should further explore the aforementioned relationships using large-scale prospective studies incorporating mechanistic approaches.

Despite these limitations, an important strength of our study is the use of saliva, a noninvasive sampling method, for biomarker analysis. The 2017 Classification of Periodontal Diseases adopted a comprehensive approach that considered both clinical and radiographic parameters as well as biological markers [32,33]. Accordingly, salivary biomarkers hold significant potential for objective and individualized assessment of disease activity [22]. The findings of this study demonstrated that leptin and calprotectin are positively associated with periodontal inflammation and tissue destruction, whereas adiponectin exhibits anti-inflammatory properties. These results suggest that salivary biomarker levels, combined with clinical parameters, may serve as potential diagnostic and monitoring tools for periodontal diseases. Furthermore, the ROC-derived cutoff values obtained in this study may represent preliminary clinical thresholds for differentiating periodontal health, gingivitis, and periodontitis. These values reflect biomarker concentrations above or below which the likelihood of periodontal inflammation and tissue destruction increases. Although these thresholds are not yet validated for diagnostic use, they provide valuable reference points that may assist clinicians in identifying individuals at higher risk and monitoring disease progression. Future longitudinal studies are required to confirm their clinical applicability.

## 5. Conclusions

This study demonstrated that elevated salivary leptin and calprotectin levels, along with decreased adiponectin levels, may be associated with the severity of periodontal disease. These findings suggest that these biomarkers could serve as biochemical indicators and potential risk markers for periodontal conditions. Further research is warranted to elucidate the role of salivary biomarkers in the complex and multifactorial pathogenesis of periodontal disease, which may contribute to early diagnosis, monitoring of disease activity, and development of individualized treatment approaches. Future studies involving patients with different stages of periodontitis are warranted to better elucidate the diagnostic and prognostic significance of salivary leptin, adiponectin, and calprotectin levels. Such studies may help establish these biomarkers as potential indicators of disease progression and treatment efficacy.

## Figures and Tables

**Table 1:** Clinical, biochemical, and demographic data.

Parameters	Periodontal Health (*n* = 55)	Gingivitis (*n* = 55)	Periodontitis (*n* = 55)	*p*-Value (ES)
Sex, *n* (%)				0.999 (0.017)
Female	27 (49.10)	27 (49.10)	28 (50.90)	
Male	28 (50.90)	28 (50.90)	27 (49.10)	
Age (years)	33 (24–38)	33 (24–38)	33 (30–38)	0.466 (0.010)
Salivary leptin (ng/mL)	2.28 (0.31–5.72) ^a^	3.03 (0.84–8.59) ^b^	4.09 (0.33–9.52) ^b^	**<0.001** (0.110)
Salivary adiponectin (ng/mL)	26.64 (0.91–52.40) ^a^	18.69 (1.55–52.48) ^a^	9.76 (0.51–46.91) ^b^	**<0.001** (0.225)
Salivary calprotectin	133.80 (7.90–411.30) ^a^	227.30 (18.80–500.10) ^b^	346.10 (38.20–570.80) ^c^	**<0.001** (0.215)
PI	0.98 (0.55–1.45) ^a^	1.65 (0.67–2.76) ^b^	2.25 (1.36–2.72) ^c^	**<0.001** (0.575)
GI	0.93 (0.27–1.57) ^a^	1.52 (0.75–2.71) ^b^	2.06 (1.28–2.71) ^c^	**<0.001** (0.603)
BOP (%)	8.80 (4.72–9.94) ^a^	26.48 (11.18–67.76) ^b^	55.70 (16.07–85.71) ^c^	**<0.001** (0.790)
PPD (mm)	1.86 (1.23–2.65) ^a^	2.17 (1.24–2.63) ^a^	3.93 (2.79–4.85) ^b^	**<0.001** (0.691)
CAL (mm)	0.75 (0.45–0.95) ^a^	0.78 (0.42–0.95) ^a^	3.50 (1.13–5.49) ^b^	**<0.001** (0.664)

Summary statistics are presented as the median and interquartile range. Group comparisons were performed using the Kruskal–Wallis test, followed by post hoc pairwise comparisons with Dunn–Bonferroni correction. Bold *p*-values indicate a statistically significant difference between groups. Identical letters in rows indicate similarity between groups, while different letters denote significant differences. ES (effect size) indicates η^2^ for Kruskal–Wallis analyses and Cramer’s V for Chi-square associations. PI, plaque index; GI, gingival index; PPD, probing pocket depth; CAL, clinical attachment loss; BOP, bleeding on probing.

**Table 2:** Diagnostic performance of salivary biomarkers for periodontal disease based on receiver operating characteristic analyses.

Groups	Biomarkers	Cut-Off	AUC [95% CI]	Sensitiviy [95% CI]	Specificity [95% CI]	*p*-Value
Healthy vs. Gingivitis	Leptin	>1.97	0.650 [0.547–0.753]	0.818 [0.691–0.909]	0.455 [0.320–0.594]	**0.004**
	Calprotectin	>192.4	0.714 [0.612–0.815]	0.691 [0.552–0.809]	0.836 [0.712–0.922]	**<0.001**
	Adiponectin	≤24.57	0.650 [0.538–0.761	0.855 [0.733–0.935]	0.636 [0.496–0.762]	**0.008**
Healthy vs. Periodontitis	Leptin	>3.269	0.733 [0.710–0.886]	0.618 [0.477–0.746]	0.836 [0.712–0.922]	**<0.001**
	Calprotectin	>223.9	0.798 [0.710–0.886]	0.709 [0.571–0.824]	0.873 [0.755–0.947]	**<0.001**
	Adiponectin	≤16.86	0.775 [0.681–0.870]	0.818 [0.691–0.909]	0.745 [0.610–0.853]	**<0.001**
Gingivitis vs. Periodontitis	Leptin	>3.835	0.619 [0.511–0.726]	0.527 [0.388–0.663]	0.782 [0.650–0.882]	**0.031**
	Calprotectin	>306.7	0.696 [0.592–0.799]	0.618 [0.477–0.746]	0.873 [0.755–0.947]	**<0.001**
	Adiponectin	≤14.04	0.795 [0.703–0.887]	0.745 [0.610–0.853]	0.873 [0.755–0.947]	**<0.001**

Receiver operating characteristic analysis was performed to evaluate the diagnostic performance of each biomarker. The cutoff values were determined based on the Youden index. The AUC, sensitivity, and specificity values are presented with corresponding *p*-values. Statistically significant *p*-values are presented in bold. AUC, area under the curve; CI: Confidence Interval.

**Table 3:** Logistic regression analysis of salivary biomarkers for differentiating periodontal disease groups.

		Univariate LR		Multiple LR		
Groups	Biomarkers	OR [95% CI]	*p*-Value	OR [95% CI]	*p*-Value	VIF
Healthy vs. Gingivitis	Leptin (Ref: ≤1.97)	3.750 [1.576–8.922]	**0.003**	2.696 [0.906–8.022]	0.075	1.046
	Calprotectin (Ref: ≤192.4)	11.425 [4.575–28.528]	**<0.001**	8.158 [2.890–23.030]	**<0.001**	1.016
	Adiponectin (Ref: >24.57)	10.281 [4.059–26.042]	**<0.001**	8.787 [3.009–25.661]	**<0.001**	1.033
Healthy vs. Periodontitis	Leptin (Ref: ≤3.269)	8.275 [3.371–20.311]	**<0.001**	7.295 [2.158–24.660]	**0.001**	1.046
	Calprotectin (Ref: ≤223.9)	16.714 [6.251–44.694]	**<0.001**	15.893 [4.552–55.486]	**<0.001**	1.101
	Adiponectin (Ref: >16.86)	13.179 [5.277–32.911]	**<0.001**	10.958 [3.306–36.319]	**<0.001**	1.057
Gingivitis vs. Periodontitis	Leptin (Ref: ≤3.835)	3.997 [1.742–9.170]	**0.001**	2.735 [0.825–9.069]	0.100	1.017
	Calprotectin (Ref: ≤306.7)	11.102 [4.244–29.042]	**<0.001**	18.313 [4.791–69.998]	**<0.001**	1.061
	Adiponectin (Ref: >14.04)	20.082 [7.399–54.503]	**<0.001**	28.246 [7.703–103.568]	**<0.001**	1.071

Univariate and multiple binary logistic regression analyses were performed to assess the association between the biomarker levels and periodontal disease status. Biomarker categories were defined based on the optimal cutoff values obtained from the receiver operating characteristic analysis. ORs with 95% CI are presented. Statistically significant *p*-values are presented in bold. OR, odds ratio; CI, confidence interval; VIF, variance inflation factor.

## Data Availability

The data that support the findings of this study are available from the corresponding author upon reasonable request. Due to ethical and privacy considerations, the raw data are not publicly available.
